# Quantification of the Human Satellite 2 (HSAT2) Repeat in the Plasma Cell-Free DNA of Patients with Colon Cancer

**DOI:** 10.3390/cimb48030256

**Published:** 2026-02-27

**Authors:** Ebru Esin Yörüker, Emre Özgür, Cemil Burak Kulle, Betül Aksu, Ilgin Gökçe Demir, Abel Bronkhorst, Stefan Holdenrieder, Ugur Gezer

**Affiliations:** 1 Department of Basic Oncology, Institute of Oncology, Istanbul University, 34093 Istanbul, Türkiye; akisik@istanbul.edu.tr (E.E.Y.); emre.ozgur.86@istanbul.edu.tr (E.Ö.); betulaksu@ogr.iu.edu.tr (B.A.); ilgingokce.demir@ogr.iu.edu.tr (I.G.D.); 2 Department of Surgery, Istanbul Medical Faculty, Istanbul University, 34093 Istanbul, Türkiye; cbkulle@istanbul.edu.tr; 3 Institute of Laboratory Medicine, German Heart Center, TUM University Hospital Munich, 80636 Munich, Germany; bronkhorst@dhm.mhn.de (A.B.); holdenrieder@dhm.mhn.de (S.H.)

**Keywords:** human satellite 2 repeat, colon cancer, liquid biopsy, DNA extraction, hybridization capture assay

## Abstract

Background/Objectives: Liquid profiling of molecular and epigenetic markers in bodily fluids is an expanding field of cancer biomarker research. Recent research activity also reveals the human satellite 2 (HSAT2) repetitive element cell-free DNA (cfDNA) as a potential cancer biomarker. Based on our recent results from targeted sequencing of HSAT2 cfDNA, we tested whether a specific HSAT2 sequence (e.g., 95 bp-HSAT2) shows greater cancer enrichment than 114 bp-SAT2, from which it derives, in patients with colon cancer. Methods: By comparing the ratio of 114 bp-HSAT2 to 95 bp-HSAT2, we investigated the increased cancer enrichment of 95 bp-HSAT2 in cfDNA samples obtained from plasma DNA extraction and a hybridization capture assay, in which HSAT2 sequences were captured from plasma using a biotin-labeled probe, in samples from colon cancer patients (*n* = 60) and polyp-controls (*n* = 60), and polyp-free controls (*n* = 60). Results: A correlation analysis between Ct values from DNA extraction and the hybridization capture assay for both 95 bp- and 114 bp-HSAT2 showed a positive correlation in patients with colon cancer and control subjects, indicating that the hybridization capture assay provides HSAT2 levels comparable to those obtained by DNA extraction. With both approaches, we found a lower 114 bp-HSAT2 to 95 bp-HSAT2 ratio in patients with colon cancer than in the control groups. The median ratio of extracted DNA was 62, 78, and 79 in patients with colon cancer, polyp-controls (*p* = 0.23), and polyp-free controls (*p* = 0.067), respectively. Capture assay values were 49, 87, and 64 in patients with colon cancer, polyp controls (*p* = 0.016), and polyp-free controls (*p* = 0.19), respectively. Even though statistical significance was not achieved in some comparisons, these results suggest that 95 bp-HSAT2 is more abundant in the blood of patients with colon cancer than 114 bp-HSAT2 in non-malignant patients. Conclusions: To our knowledge, this is the first study to conduct a hybridization capture assay using a biotinylated probe as a feasible approach for targeted enrichment of cfDNA from plasma. Our results confirm the outcomes of our recent article based on targeted sequencing and reveal that some specific HSAT2 sequences may exhibit increased cancer abundance.

## 1. Introduction

Colon cancer is the third most common cancer, with approximately 1.9 million new cases each year [1]. It is the second leading cause of cancer-related deaths worldwide, with approximately 935,000 deaths each year. The development of biomarkers for the screening and early detection of colon cancer is crucial. Although numerous blood-based markers have been proposed for colon cancer screening, only a few have been translated into clinical practice [2], highlighting the challenge of establishing biomarker clinical utility, which involves complex processes of validation and implementation in routine medical settings. The expanding field of cancer biomarkers involves the liquid profiling of molecular and epigenetic markers in whole blood, serum, plasma, urine, or other biological fluids, rather than in tissue. The analysis of tumor-derived fraction (e.g., circulating tumor-DNA, ctDNA) of cell-free DNA (cfDNA) in plasma is one of the main applications of liquid profiling, which is increasingly gaining ground in oncology clinical practice to support cancer detection and diagnosis, assess prognosis, enable treatment monitoring, and improve patient follow-up [3].

**Figure 1:**
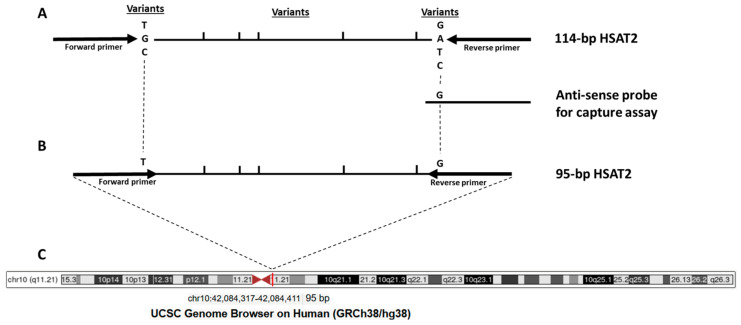
Schematic representation of the target HSAT2 region. (**A**) 114 bp-HSAT2 amplicon with the location of variant nucleotides; (**B**) Newly designed 95 bp-HSAT2 with primer binding sites relative to the 114 bp-HSAT2; (**C**) The chromosomal location of 95 bp-HSAT2 in the pericentromeric region of chromosome 10.

Pericentric satellites (HSATs) are tandemly repeated DNA sequences in the human genome with four subtypes, HSATI, HSATII, HSATIII, and HSATIV, located in the pericentric heterochromatin on a subset of human chromosomes, such as chromosomes 1, 10, and 16. Recent research has highlighted the importance of pericentric satellites, such as HSAT2, for tumorigenesis and their potential as biomarkers. Two initial papers, published in 2009 and 2011, reported the overexpression of pericentromeric satellite repeat RNAs in epithelial cancers [4,5]. In 2015, Bersani et al. reported increased HSAT2 copy numbers in tumor cells by whole-genome sequencing. They also showed that the expansion of HSAT2 sequences in tumor cells is mediated by the insertion of RNA-derived DNA sequences into the pericentromeric regions [6]. As a result of increased expression in the tumor being reflected in the circulation, significantly increased levels of HSAT2 RNA sequences were found in the sera of pancreatic cancer patients compared to controls [7]. Pericentric satellites are the most overrepresented sequences in plasma DNA under physiological conditions (e.g., in healthy individuals) relative to their proportions in the human genome, as determined by whole-genome sequencing [8], making HSAT2 an appropriate candidate biomarker for liquid profiling. After demonstrating that HSAT2 sequence levels are higher in the plasma DNA of cancer patients, including colon cancer, compared to healthy subjects [9], our subsequent targeted sequencing study showed that some variant sequences of 114 bp HSAT2 fragments (e.g., chr10: 42084257–42084497; Figure 1A) are more common in the plasma cfDNA of breast cancer patients than other 114 bp HSAT2 sequences compared to samples from healthy controls, likely a consequence of their chromosomal location [10]. The biological value of HSAT2 sequence variants and their clinical impact on tumor development need to be determined. Furthermore, since cfDNA fragments derived from tumor cells are more fragmented than those derived from non-tumoral cells, testing shorter HSAT2 sequences in clinical samples could be more informative. Therefore, we derived a shorter sequence from the 114 bp-HSAT2, in which variant bases with higher cancer abundance were covered by the primer binding sites (Figure 1B), resulting in the 95 bp-HSAT2, which was annotated to the pericentromeric region of chromosome 10 (Figure 1C). In the present study, we hypothesize that 95 bp-HSAT2 shows greater cancer enrichment than parental 114 bp-HSAT2.

## 2. Materials and Methods

### 2.1. Samples and Study Design

Samples were collected from three types of participants: the first group comprised patients (*n* = 60; 34 males, 26 females; median age 65) with pathologically confirmed non-metastatic colon cancer. Fifty-three percent of the patients had stage III-IV disease, while the remaining patients suffered from stage I-II colon cancer (Table 1). The second group consisted of individuals who had no tumor, but nonadenomatous polyps in their colon (the polyp controls, *n* = 60, median age 58, 38 males and 22 females), while the third group included individuals with no cancer or polyps in their colons (the polyp-free controls, *n* = 60, median age 56, 40 males and 20 females). Blood samples were withdrawn before the colonoscopy. The study was conducted with the approval of the Ethics Committee of Istanbul Medical Faculty, and all participants provided informed consent (the Ethics Committee approval number 2023/2009).

We tested the enhanced cancer enrichment of 95 bp-HSAT2 compared to 114 bp-HSAT2 using two approaches: in the first, we amplified 95 bp-HSAT2 and 114 bp-HSAT2 from plasma cfDNA isolated with a standard cfDNA extraction kit. In the second approach, we performed a hybridization-based capture assay using a biotin-labeled oligonucleotide probe to capture and pull down HSAT2 sequences from plasma (Figure 1), followed by PCR amplification of 95 bp-HSAT2 and 114 bp-HSAT2. We then compared the ratio of 114 bp-HSAT2 to 95 bp-HSAT2 between patients with colon cancer and the control groups, where a lower ratio in the cancer group indicates a higher enrichment of 95 bp-HSAT2 relative to 114 bp-HSAT2.

### 2.2. DNA Extraction from Plasma

Blood samples were collected into K2-EDTA tubes and centrifuged at 1100× *g* for 20 min within 0–4 h of collection. The plasma fraction was transferred to new tubes and centrifuged again to remove any remaining cells or debris. Plasma samples were stored at −80 °C until use. Plasma DNA isolation was performed using a commercial kit (QIAamp Circulating DNA Kit, Qiagen GmbH, Hilden, Germany) according to the manufacturer’s instructions. Two hundred μL of plasma was used. After DNA quantification using the Nanodrop spectrometer (Thermo Fisher Scientific, Wilmington, NC, USA), the extracted DNA samples were stored at −20 °C. The mean plasma DNA concentrations in patients with colon cancer and polyp- and polyp-free controls were 43, 47, and 33 ng/mL, respectively.

### 2.3. Hybridization Capture Assay

To capture and pull down HSAT2 sequences from plasma samples, we performed a hybridization capture assay employing a biotin-labeled antisense probe specific for putative cancer-enriched HSAT2 sequences (Figure 1), using a modified protocol from Oreskovic and Lutz [11]. Streptavidin-conjugated beads served to isolate the biotinylated oligonucleotide-target sequence complexes.

The first step of the assay was immobilizing the capture probe on magnetic beads. To do this, magnetic beads were vortexed to ensure homogeneous dispersion. Approximately 5 µL of beads per 1 mL of plasma was pipetted into a tube. The beads were then washed three times with high-salt buffer (1 M NaCl, 10 mM Tris-HCl, pH 8, 0.05% Tween-20) and placed on a magnetic rack, allowing them to settle for 1 min. The supernatant was removed. After washing, the beads were dissolved again in high-salt buffer, and 5 pmol of hybridization probe was added. The sequence of the probe was 5′-TGCGATTCCATTAGATGATGACTCCTTTC-3′. The probes were allowed to bind to the magnetic beads on a rocking platform for 15 min at RT. The settled beads were washed three times with high-salt buffer. Magnetic beads without added probes were used as the control. In the next step, magnetic beads (5 µL) with labeled probe in the presence of 1 M NaCl and 0.1% (*v*/*v*) Tween 20 were mixed with the plasma sample and incubated at 90 °C for denaturation for 15 min. After denaturation, the hybridization was achieved by incubating the mixture on a rocking platform for 30 min. The beads were then settled by centrifugation at 5000× *g* for 5 min and washed three times with high-salt buffer. Finally, freshly prepared 2 mM NaOH was added to the beads to extract the target HSAT2 sequences hybridized with the biotin-labeled probe. The supernatant containing the cfDNA fragments (approximately 20–22 µL) was transferred to a new tube, and the mixture was neutralized by adding 3.5 µL of 100 mM HCl. The captured DNA was then purified using a commercial kit according to the instructions.

### 2.4. Real-Time PCR

Real-time PCR was conducted using dye-based PCR amplification in a LightCycler 480 PCR (Roche Diagnostics, Basel, Switzerland) instrument using a Power SYBR Green PCR Master Mix (Life Technologies, Carlsbad, CA, USA) according to the instructions at an annealing temperature of 58 °C. Primers used for 95 bp- and 114 bp-HSAT2 are displayed in Table 1. We performed two PCR measurements, which also included duplicates of all samples. The mean ratio of 114 bp-HSAT2 to 95 bp-HSAT2 was calculated using the formula 2^ΔCt^.

### 2.5. Measurement of Tumor Markers

Carcinoembryonic antigen (CEA) and carbohydrate antigen 19-9 (CA19-9) are the most commonly used tumor markers in the clinical evaluation of patients with colon cancer [12]. We measured pretreatment serum levels of CEA and CA19-9 as previously described [13]. Since a CEA level above 5 ng/mL at the time of initial colon cancer diagnosis indicates a poor prognosis [14], we set this concentration as the cutoff. For CA19-9, levels around 35 U/mL are associated with shorter survival in colon cancer patients [15]; we used this value as the cutoff. We investigated the ratio of 114 bp-HSAT2 to 95 bp-HSAT2 in patients below and above the cutoff value of CEA or CA-19-9.

### 2.6. Statistics

Statistical analyses and figure drawings were performed using the statistical tool GraphPad (version 10.6.1). We used the Shapiro–Wilk test to determine whether HSAT2 cfDNA levels were normally distributed in the study groups and found a non-normal distribution. The Kruskal–Wallis test was used to determine whether HSAT2 levels differed between the study groups (the patients, polyp controls, and polyp-free controls). We used the Mann–Whitney U test to compare the study groups and to examine the correlations between plasma HSAT2 levels and clinicopathological factors. A *p*-value < 0.05 was considered statistically significant.

## 3. Results

**Figure 2:**
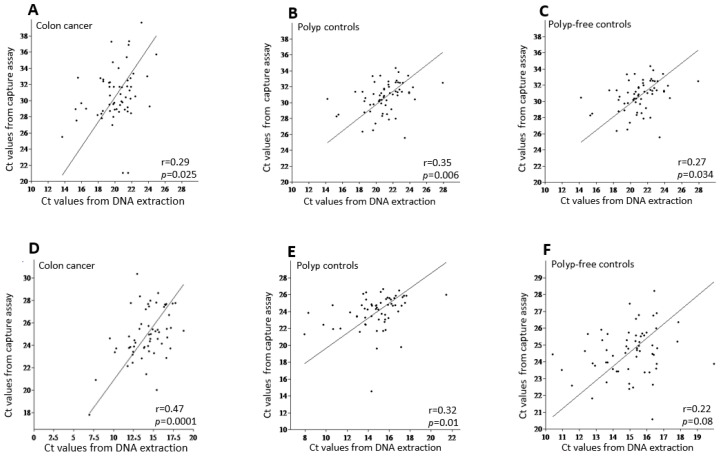
The correlation analysis between the mean Ct values of DNA extraction vs. the hybridization capture assay for 95 bp-HSAT2 (upper panel, (**A**–**C**)) and 114 bp-HSAT2 (lower panel, (**D**–**F**)) in the study groups. r, the Pearson correlation coefficient.

We first investigated whether the hybridization capture assay yields results comparable to those of the conventional DNA extraction method. To this end, we analyzed the correlation between Ct values from DNA extraction and the hybridization capture assay for both 95 bp- and 114 bp-HSAT2 (Figure 2). We found a positive, largely statistically significant, correlation between Ct values of DNA extraction and the hybridization capture assay in both the cancer patients and control groups. The upper panel (A–C) in Figure 2 shows the correlation for the 95 bp-HSAT2, while the lower panel (D–F) illustrates the correlations for the 114 bp-HSAT2. These findings indicate that the hybridization capture assay yields comparable amounts of HSAT2 to the conventional DNA extraction method.

**Figure 3:**
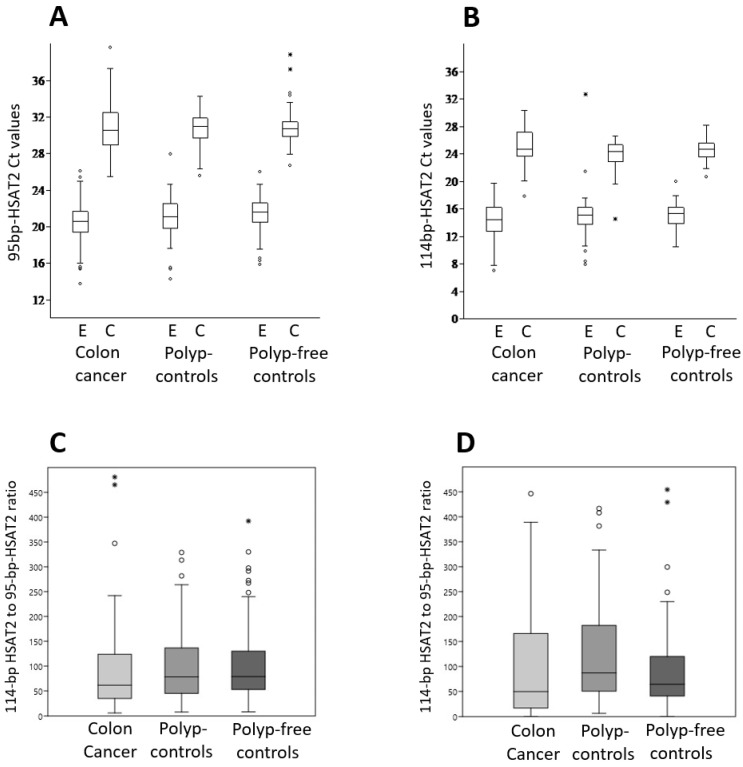
HSAT2 amplification in the study groups. The 95 bp-HSAT2 and 114 bp-HSAT2 were amplified from cfDNA samples extracted from plasma using a kit or captured by biotin-labeled antisense oligonucleotides. Ct values for 95 bp-HSAT2 (**A**) and 114 bp-HSAT2 (**B**) within the study groups, as determined by the DNA extraction (E) and hybridization capture assay (C); (**C**) the ratio of 114 bp-HSAT2 to 95 bp-HSAT2 obtained via DNA extraction in colon cancer patients and control groups; (**D**) The ratio of 114 bp-HSAT2 to 95 bp-HSAT2 obtained via hybridization capture assay in patients with colon cancer and control groups. The box plots show the median values, the interquartile range (25 and 75%) and the maximum and minimum values.

Figure 3A,B show the distribution of Ct values for the 95 bp-HSAT2 and 114 bp-HSAT2 within the study groups as determined by the DNA extraction and hybridization capture assay. As expected, the 95 bp-HSAT2 is less abundant (e.g., higher Ct values) than the 114 bp-HSAT2, as it represents a specific sequence, whereas the primers for 114 bp-HSAT2 cover several alternative sequences (see Figure 1) and some longer ones. The variance in Ct values was higher in the DNA extraction than in the capture assay. It was highest in patients with colon cancer compared to control groups, suggesting a greater oscillation of cfDNA concentrations in cancer patients.

We then compared the ratio of 114 bp-HSAT2 to 95 bp-HSAT2 between the patients with colon cancer and control subjects for both the extracted DNA and the hybridization capture assay. Both methods revealed a decreased, partially statistically significant ratio of 114 bp-HSAT2 to 95 bp-HSAT2 in the patients with colon cancer compared to the control groups (Figure 3B,C). The median 114 bp-HSAT2 to 95 bp-HSAT2 ratios from isolated DNA were 62 (95% confidence interval (CI): 44.4–84.7), 78 (95% CI: 60.6–101.2), and 79 (95% CI: 64.9–100), respectively, in colon cancer patients, polyp controls (*p* = 0.23), and polyp-free controls (*p* = 0.067). The hybridization capture assay values were 49 (95% CI: 32.6–93.5), 87 (95% CI: 66.7–135.7), and 64 (95% CI: 56.2–78.1), respectively, in patients with colon cancer, polyp controls (*p* = 0.016), and polyp-free controls (*p* = 0.19). Although a cautious interpretation is required in some comparisons due to the lack of statistical significance, these results suggest that 95 bp-HSAT2 is more abundant in the blood of patients with colon cancer compared to non-malignant patients than 114 bp-HSAT2.

Finally, we examine any links between plasma relative 95 bp-HSAT2 levels (e.g., the ratio of 114 bp-HSAT2 to 95 bp-HSAT2) and clinopathological parameters of colon cancer patients (Table 2). We identified a statistically significant link between plasma 95 bp-HSAT2 levels from hybridization capture assay and CA19-9 concentrations (*p* = 0.01) and tumor localization (e.g., right colon) (*p* = 0.008). Higher 95 bp-HSAT2 levels (e.g., a lower 114 bp-HSAT2 to 95 bp-HSAT2 ratio) from DNA extraction were associated with male gender (*p* = 0.005). However, these findings should be interpreted cautiously because the number of patients in the subgroups is small. On the other hand, we investigated whether the ratio of 114 bp-HSAT2 to 95 bp-HSAT2 might be clinically relevant for distinguishing early-stage diseases from benign conditions. To this end, we compared samples from stages I and II with polyp controls. The results showed that the ratio differed only in the samples obtained by capture assay between patients with stage I and II diseases (median 40) and polyp controls (median 87; *p* = 0.05), while the difference was considerably smaller in the samples obtained by DNA extraction (median 66.6 vs. 78, respectively; *p* = 0.23).

## 4. Discussion

Colon cancer is a malignant disease for which extensive biomarker research has been conducted. The research mainly focused on the diagnostic and prognostic value of cfDNA, ctDNA, and methylated DNA in the plasma of patients with colon cancer. The potential of cfDNA to revolutionize the screening, detection, and prognosis of colon cancer lies in overcoming several challenges, including standardizing pre- and post-analytical variables and developing technologies with high sensitivity and specificity. As a result of intensive research, a few biomarkers have been developed, such as methylated septin 9 in plasma DNA [16]. The present study was based on our previous results to assess the increased cancer enrichment of a specific HSAT2 sequence derived from the 114 bp-HSAT2, as determined by targeted sequencing of HSAT2 amplicons [10] (Figure 1). With two different approaches, DNA extraction and hybridization capture assay, we investigated increased cancer enrichment of 95 bp-HSAT2 compared to 114 bp-HSAT2.

The reason for using a hybridization capture assay in our study was to specifically enrich the target sequence HSAT2 from plasma. Hybridization capture assays are a technique for selectively enriching target DNA/RNA sequences from a sample and are currently widely used in next-generation sequencing. A longer probe compared to the PCR primer increases specificity. Biotinylated antisense DNA probes are used in biomedical research for various purposes, such as the identification of DNA-binding proteins, the enrichment of specific RNA molecules, the identification of RNAs in direct RNA-RNA interactions with non-coding RNAs, and the investigation of ribonucleoprotein complex assembly [17,18,19,20]. We adapted the protocol of Oreskovic A and Lutz BR for plasma [11], who used the hybridization capture assay to enrich short cfDNA fragments from large urine volumes.

Since we were able to demonstrate a positive, albeit weak, correlation between the Ct values of HSAT2 (both 95 bp and 114 bp) from cfDNA extraction and the hybridization capture assay, this shows that the hybridization capture assay provides comparable and therefore reliable results as DNA extraction, even though the yield was considerably lower (see Figure 3A,B). The weak correlation between cfDNA extraction and the hybridization capture assay, as well as the lower yield from the latter, could be explained by the fact that the conventional cfDNA extraction method and probe-based hybridization capture are fundamentally different processes; therefore, a strong quantitative correlation in yield should not be expected. Standard extraction methods recover total cf DNA in a largely sequence-independent manner, including all HSAT2 fragments. In contrast, biotin-labeled probe capture depends on sequence-specific hybridization, so only fragments that bind efficiently to the probe are retained. Sequence heterogeneity within HSAT2 repeats, partial mismatches, secondary structure formation, similarity to other satellite sequences, short fragment length, and stringent wash steps can all reduce capture efficiency. Because HSAT2 consists of highly repetitive and structurally complex sequences within fragmented cfDNA, lower recovery after capture likely reflects technical limitations of enrichment rather than true biological depletion.

Regardless of the extraction/enrichment method used to obtain cfDNA from plasma, the primary challenge is normalizing plasma levels of the target sequence. This is due, firstly, to the high variability between samples from different individuals (see Ct values in Figure 3A,B) resulting from differing total cfDNA concentrations, and secondly, to the fact that most of the cfDNA does not originate from tumor cells [21]. Not only are there interindividual differences, but cfDNA concentrations also fluctuate within samples from the same individual, even when collected throughout the day or on different days, which affects the cfDNA yield [22,23]. Appropriate genes or sequences for normalizing the levels of target genes/sequences within the cfDNA fragment population are required but lacking. Since we used a hybridization capture assay to enrich for a specific HSAT2 sequence, no other sequences were suitable for normalization. Therefore, we considered the ratio of 114 bp-HSAT2 to 95 bp-HSAT2 as a measure of the normalization and enrichment of 95 bp-HSAT2 in the plasma of cancer patients. In cfDNA samples obtained from DNA extraction and the capture assay, we found a lower 114 bp-HSAT2 to 95 bp-HSAT2 ratio in patients with colon cancer than in controls, which included individuals with polys and no polys. The aforementioned ratio was 25–27% lower in the patient group than in the control groups for DNA extraction and 30–43% lower for the capture assay. Although we emphasize the need for cautious interpretation due to the lack of statistical significance in some comparisons, these results suggest that a low-copy-specific sequence (e.g., 95 bp-HSAT2) shows higher cancer enrichment in the plasma of patients with colon cancer than 114 bp-HSAT2. It should be noted that plasma levels of the 114 bp-HSAT2 sequences, normalized to LINE1, were also higher in cancer patients than in healthy subjects [9], which could explain the lack of statistical significance in some comparisons between patients and controls in the present study. On the other hand, many pre-analytical and analytical procedures for cfDNA analysis are not standardized [24,25], so numerous analytical variables could also have influenced the results of our study. For example, it is well documented that extraction kits from different brands produce different yields of plasma cfDNA [26,27]. The extent of cfDNA fragmentation can also influence the results of DNA extraction and hybridization capture assays, as it cannot be ruled out that some short DNA fragments may be lost during DNA extraction, the capture assay washing steps, and subsequent DNA cleaning.

This study on the clinical relevance of 95 bp-HSAT2 in the plasma of patients with colon cancer differs significantly from our previous work. In the previous study, we amplified HSAT2 using primers that generated multiple amplicons from the plasma of cancer patients and quantified [9] or sequenced them [10]. In contrast, this study focuses on a specific, rare sequence and shows that it may be more highly enriched in cancer cells. We suppose that the increased cancer enrichment of the 95 bp-HSAT2 could be a result of its chromosomal localization (Figure 1C). The selection of this 95 bp-HSAT2 sequence leads to the exclusion of many HSAT2 sequences from 114 bp-HSAT2, as these sequences may be located on several chromosomes, including chr1, chr10, and others. We showed that HSAT2 sequences originating from primers that more strongly amplify chromosome 10 were higher in cancer patients than those originating from chromosome 1 [9]. This differential abundance of some HSAT2 sequences could be related to the expansion mechanism of the HSAT2 repetitive element in tumor cells, as HSAT2 sequences have been shown to expand via an RNA-derived DNA intermediate that inserts into pericentromeric regions [6]. This expansion in copy numbers may be more pronounced for some regions of pericentromeres or chromosomes.

## 5. Conclusions

To our knowledge, this is the first study to conduct a hybridization capture assay using a biotinylated probe to capture cfDNA from plasma and compare it with DNA extraction. A positive correlation between Ct values from the DNA extraction and the hybridization capture assay indicates that the hybridization capture assay provides comparable results. In cfDNA samples obtained from DNA extraction and the capture assay, we found a lower 114 bp-HSAT2 to 95 bp-HSAT2 ratio in patients with colon cancer than in controls. Thus, our results confirm the outcomes of our recent article based on targeted sequencing and reveal that targeted enrichment is feasible, and that some specific HSAT2 sequences may exhibit increased cancer abundance. These results need to be confirmed in independent cohorts and across different cancer types. Furthermore, elucidating the biological and clinical significance of specific HSAT2 sequences in cancer is necessary. Future studies could also investigate technical aspects, such as the analysis of fragment size distributions, testing probes or hybridization conditions with different stringency, comparing different amplicon lengths, or applying orthogonal quantification methods such as short amplicon qPCR or digital PCR.

## Figures and Tables

**Table 1:** Primer and probe sequences used.

The Target HSAT2	Sequence
95 bp-HSAT2	5′-CACTTCCGTTCAATTATTCC-3 5′-GTCATCATCTAATGGAATCG-3′
114 bp-HSAT2	5′-GACGATTCCATTCACTTCC-3′ 5′-GAAAGGAGTCATCATCTAATGG-3′
Probe sequence	5′-GCGATTCCATTAGATGATGACTCCTTTC-3′.

**Table 2:** Association of the 114 bp-HSAT2 to 95 bp-HSAT2 ratio with clinicopathological characteristics.

Variable	DNA Extraction			Hybridization Capture Assay		
	No. of Patients	Median	*p* Value	No. of Patients	Median	*p* Value
Age			0.47			0.72
≥60	37	72		36	97	
<60	23	112		22	93	
Gender			0.005			0.42
Male	34	62		32	91	
Female	26	117		26	96	
CEA (ng/mL)			0.98			0.44
≤5	37	108		36	89	
>5	21	111		20	57	
CA 19-9 (U/mL)			0.28			0.01
≤35	48	117		46	93	
>35	10	62		10	21	
Tumor size (cm)			0.25			0.29
≥5	25	55		24	53	
<5	34	108		33	96	
Lymphatic metastasis			0.88			0.92
N0	26	109		24	88	
N-N3	33	117		33	91	
TNM Stage			0.73			0.74
I-II	26	99		24	93	
III-IV	32	112		32	89	
Tumor localization			0.43			0.008
Right	21	108		21	47	
Left	39	0.01		37	97	
Venous invasion			0.89			0.55
No	27	104		26	78	
Yes	32	100		31	88	
Crohn’s like			0.28			0.18
No	10	72		10	0.03	
Yes	49	99		47	33	
Lymphocyte infiltration			0.13			0.12
No	11	108		11	206	
Yes	48	96		46	51	
Perineural invasion			0.36			0.25
No	27	66		25	48	
Yes	32	96		32	93	

## Data Availability

The raw data supporting the conclusions of this article will be made available by the authors upon request.
